# Supplementary material for: Gadolinium-enhanced MRI visualizing backflow at increasing intra-renal pressure in a porcine model
Source: PLoS One. 2023 Feb 16;18(2):e0281676. doi: 10.1371/journal.pone.0281676 (PMC9934347; doi:10.1371/journal.pone.0281676)
Supplement: S1 Table — Hematoxylin eosin (H&E) and periodic acid-shiff (PAS) staining of kidney tissue samples. Morphology was evaluated by standard light microscopy. (DOCX) [file pone.0281676.s001.docx]

| Animal | Side | Sample location | Inflammation | Acute tissue damage |
| --- | --- | --- | --- | --- |
| 1 | Left | Upper kidney | No | No |
| 1 | Left | Mid kidney | No | No |
| 1 | Left | Lower kidney | No | No |
| 1 | Right | Upper kidney | No | No |
| 1 | Right | Mid kidney | No | No |
| 1 | Right | Lower kidney | No | No |
| 2 | Left | Upper kidney | No | No |
| 2 | Left | Mid kidney | No | No |
| 2 | Left | Lower kidney | No | No |
| 2 | Right | Upper kidney | No | No |
| 2 | Right | Mid kidney | No | No |
| 2 | Right | Lower kidney | No | No |
| 3 | Left | Upper kidney | No | No |
| 3 | Left | Mid kidney | No | No |
| 3 | Left | Lower kidney | No | No |
| 3 | Right | Upper kidney | No | No |
| 3 | Right | Mid kidney | No | No |
| 3 | Right | Lower kidney | No | No |
| 4 | Left | Upper kidney | No | No |
| 4 | Left | Mid kidney | No | No |
| 4 | Left | Lower kidney | No | No |
| 4 | Right | Upper kidney | No | No |
| 4 | Right | Mid kidney | No | No |
| 4 | Right | Lower kidney | No | No |
| 5 | Left | Upper kidney | No | No |
| 5 | Left | Mid kidney | No | No |
| 5 | Left | Lower kidney | No | No |
| 5 | Right | Upper kidney | No | No |
| 5 | Right | Mid kidney | No | No |
| 5 | Right | Lower kidney | No | No |
